# Supplementary material for: Intracellular morphogenesis of diatom silica is guided by local variations in membrane curvature
Source: Nat Commun. 2024 Sep 10;15:7888. doi: 10.1038/s41467-024-52211-x (PMC11385223; doi:10.1038/s41467-024-52211-x)
Supplement: Supplementary file 1 — Supplementary Information [file 41467_2024_52211_MOESM1_ESM.pdf]

Supplementary Information

Intracellular morphogenesis of diatom silica is guided by local variations in membrane curvature

Lior Aram, Diede de Haan, Neta Varsano, James B. Gilchrist, Christoph Heintze, Ron Rotkopf, Katya Rechav, Nadav Elad, Nils Kröger, Assaf Gal*

*Corresponding author. Email: assaf.gal@weizmann.ac.il

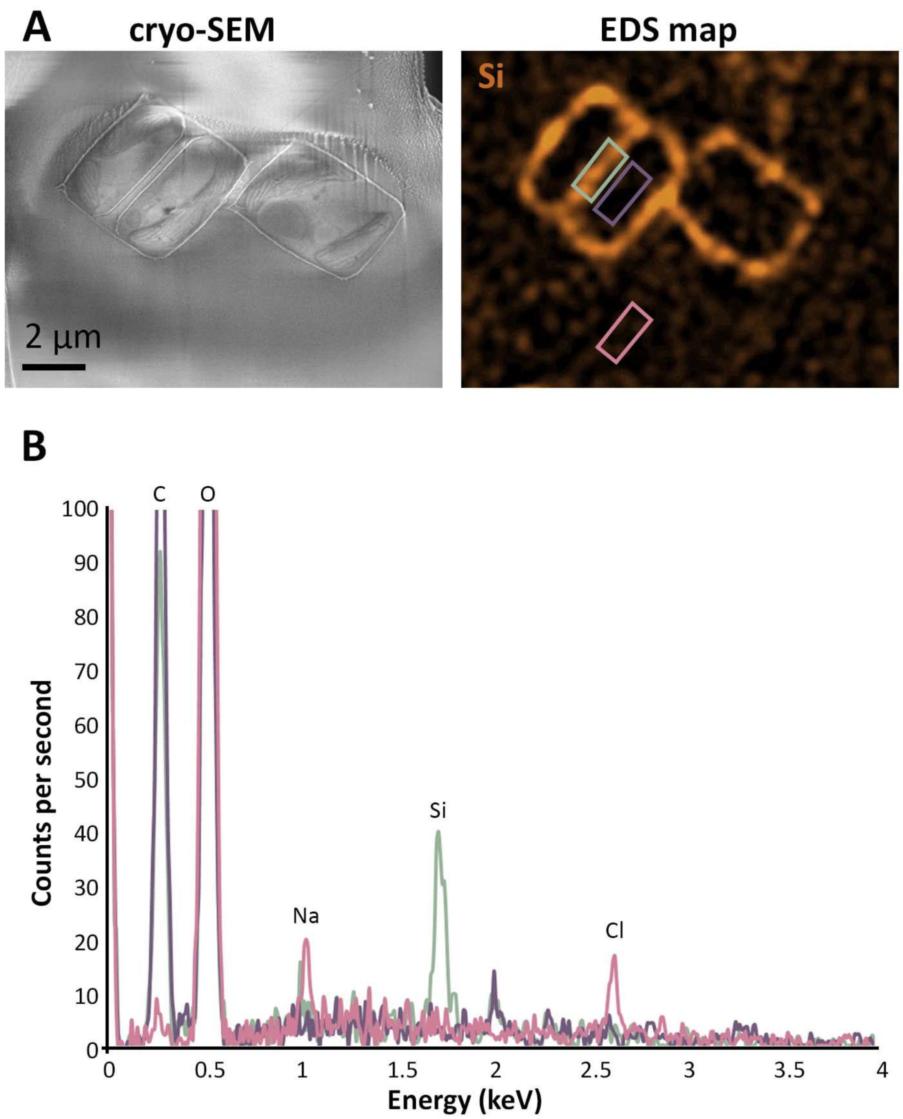

Figure S1 – Intracellular cryoEDS measurements showing silicon signal in the SDV location. A) cryoSEM and EDS map of one dividing and one not dividing *T. pseudonana* cell. B) EDS spectra at the different regions of the map, as indicated by the rectangles in A. Source data are provided as a Source Data file.

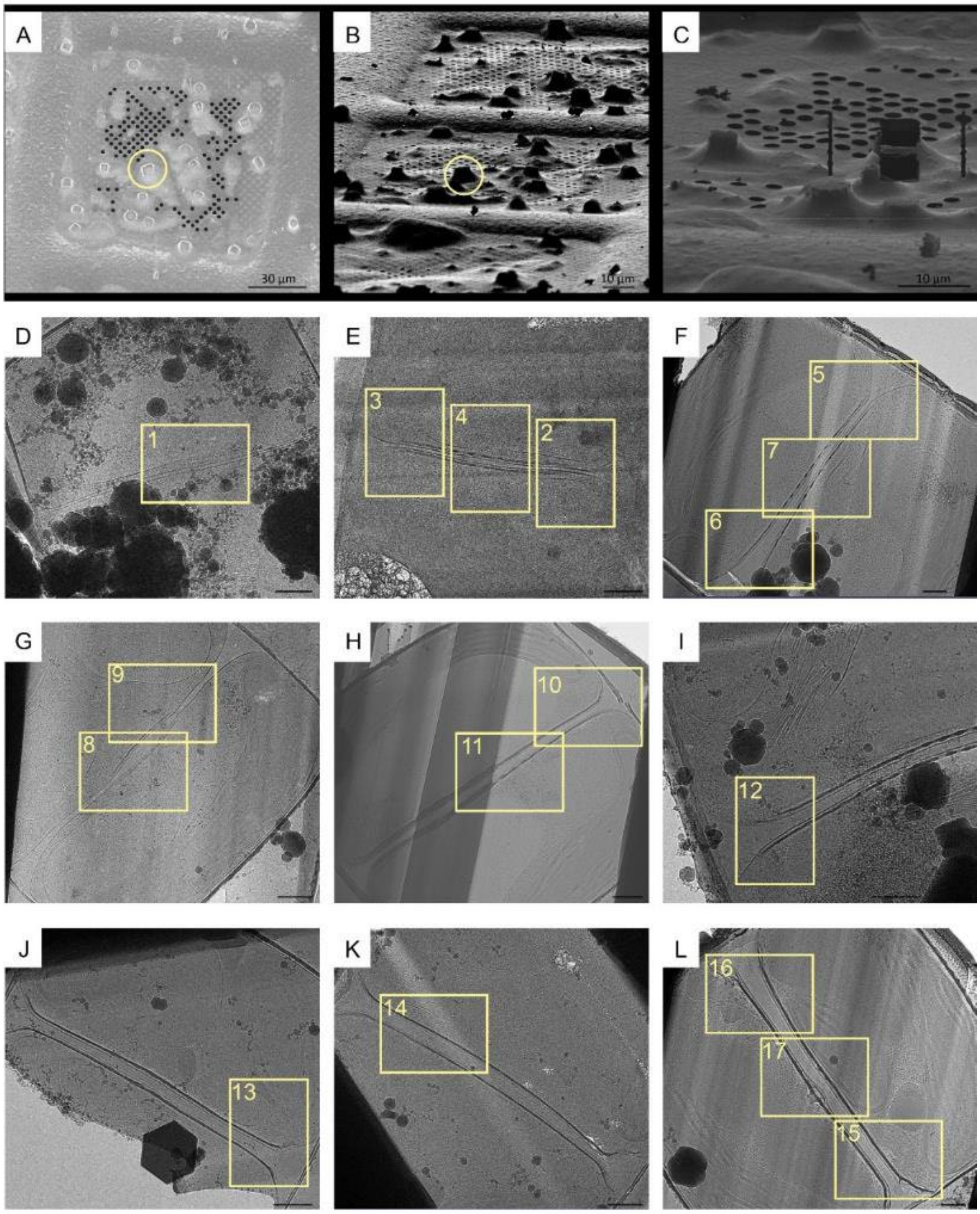

Figure S2 – Overview of lamella preparation and low magnification images of lamellae. Plate 1 out of 2. A) cryoSEM image of vitrified *T. pseudonana* cells on a TEM grid. B) cryoFIB image of the vitrified cells. The yellow circle in panels A and B shows the same cell in the preferred geometry of lamella preparation. C) high magnification cryoFIB image shows the rectangles of the rough milling. D-L) Low-magnification images of the lamellae used for data collection. The cells are ordered according to their developmental stages. Yellow rectangles show the area of the acquired tomography data and are numbered according to their number in Fig. S3. Scale bars D-L are 500 nm.

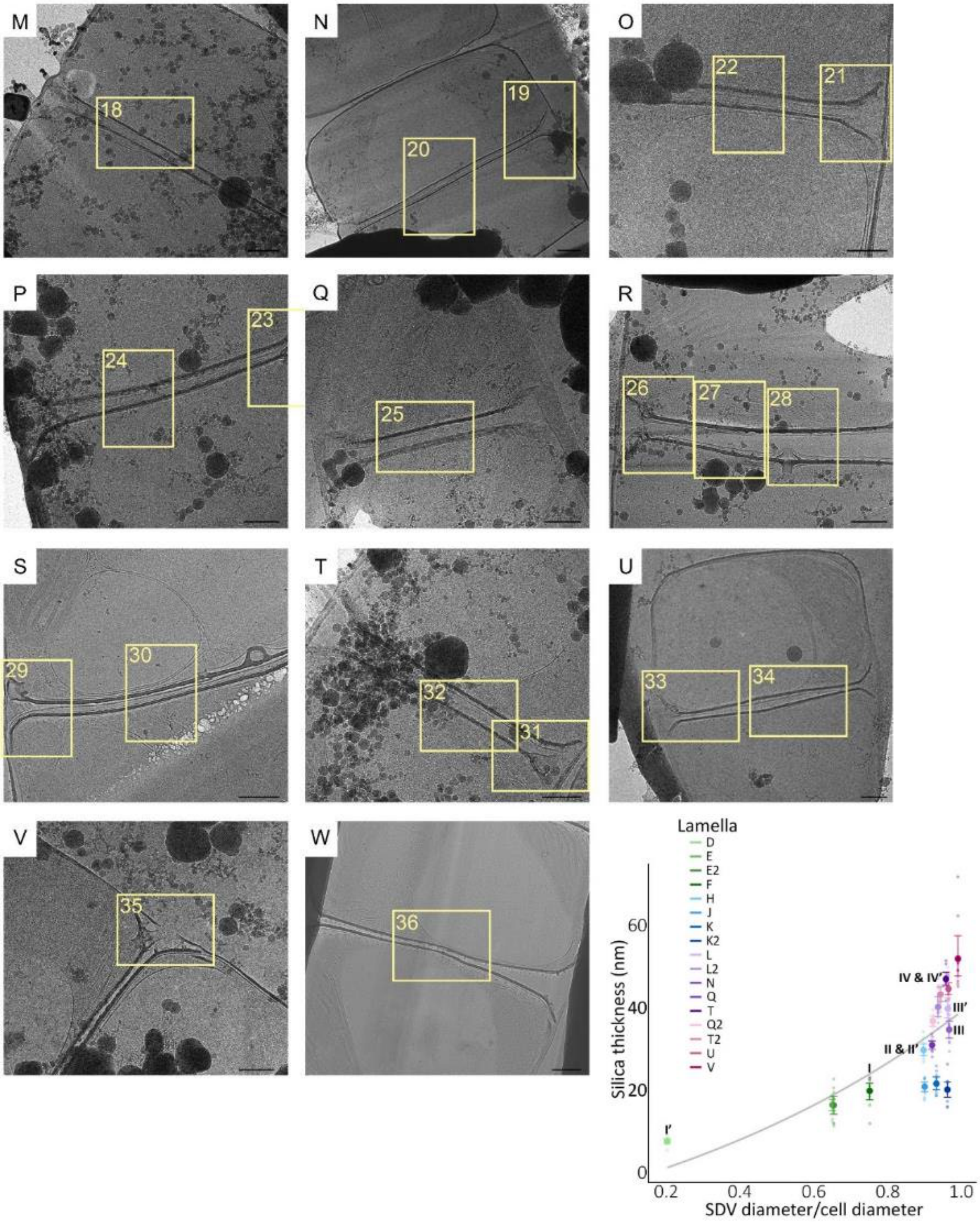

Figure S2 –Plate 2 out of 2.

M-W) continued from Plate 1. The graph at the end shows that silica thickness increases exponentially with the maturation stage ($p<0.0001$). This correlation, together with specific inspection of the silica architecture, was used to reconstruct the developmental timeline of the SDVs in the different cells (labels are identical in the graph and the panels). Scale bars in M-W is 500 nm. Source data are provided as a Source Data file.

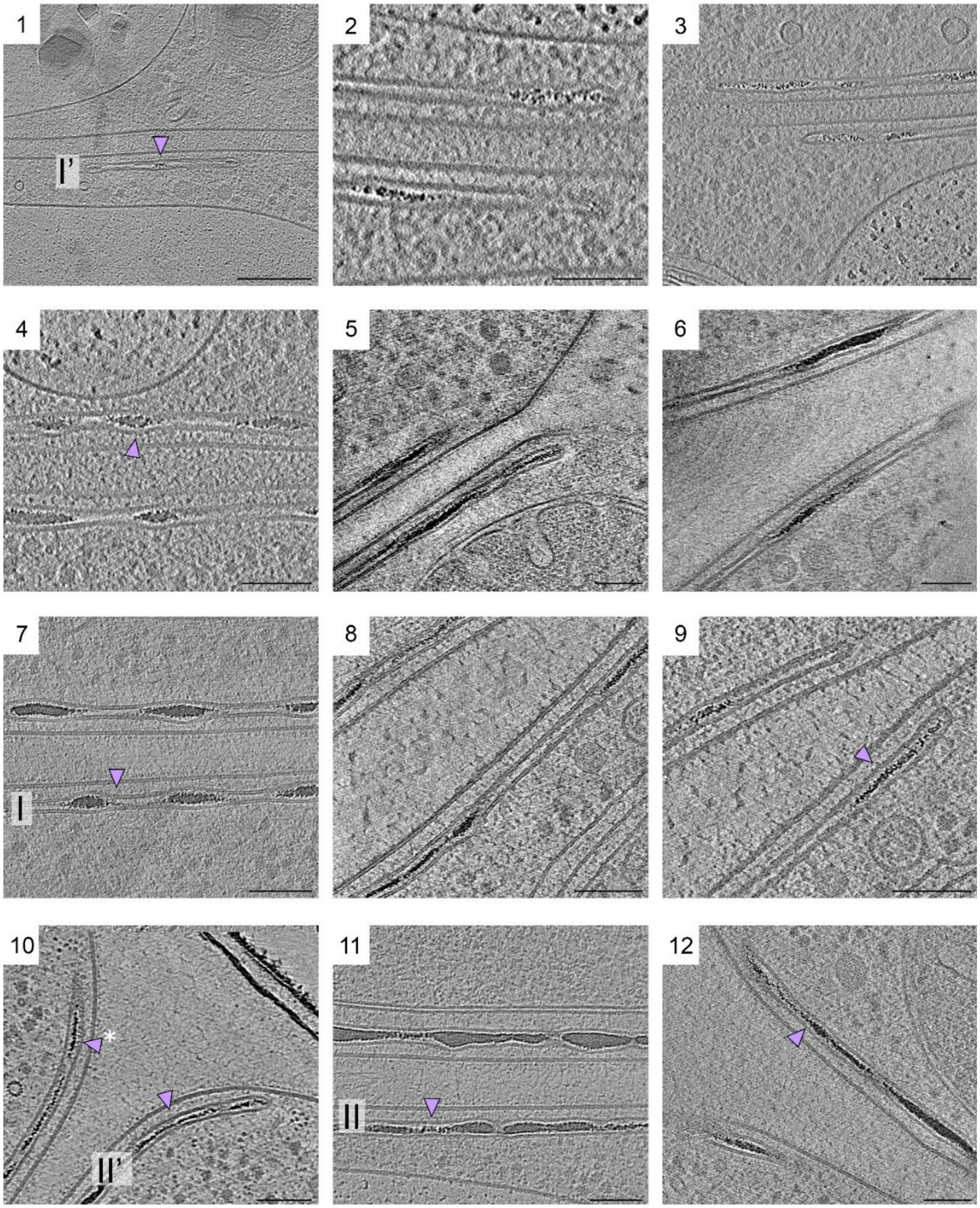

Figure S3 – Overview of the complete datasets that contain forming SDVs. Plate 1 out of 3.

A representative slice from each of the 34 datasets that contain an SDV is presented in each panels 1-34. Datasets 35 and 36 were used for silica thickness measurements for Fig. S2. Scale bars are 100 nm. Datasets that were used in the main figures are indicated with the same Roman numbers (with and without tags) Purple arrowheads indicate datasets that were analyzed in Fig. 3 (See Plate 3 for a legend).

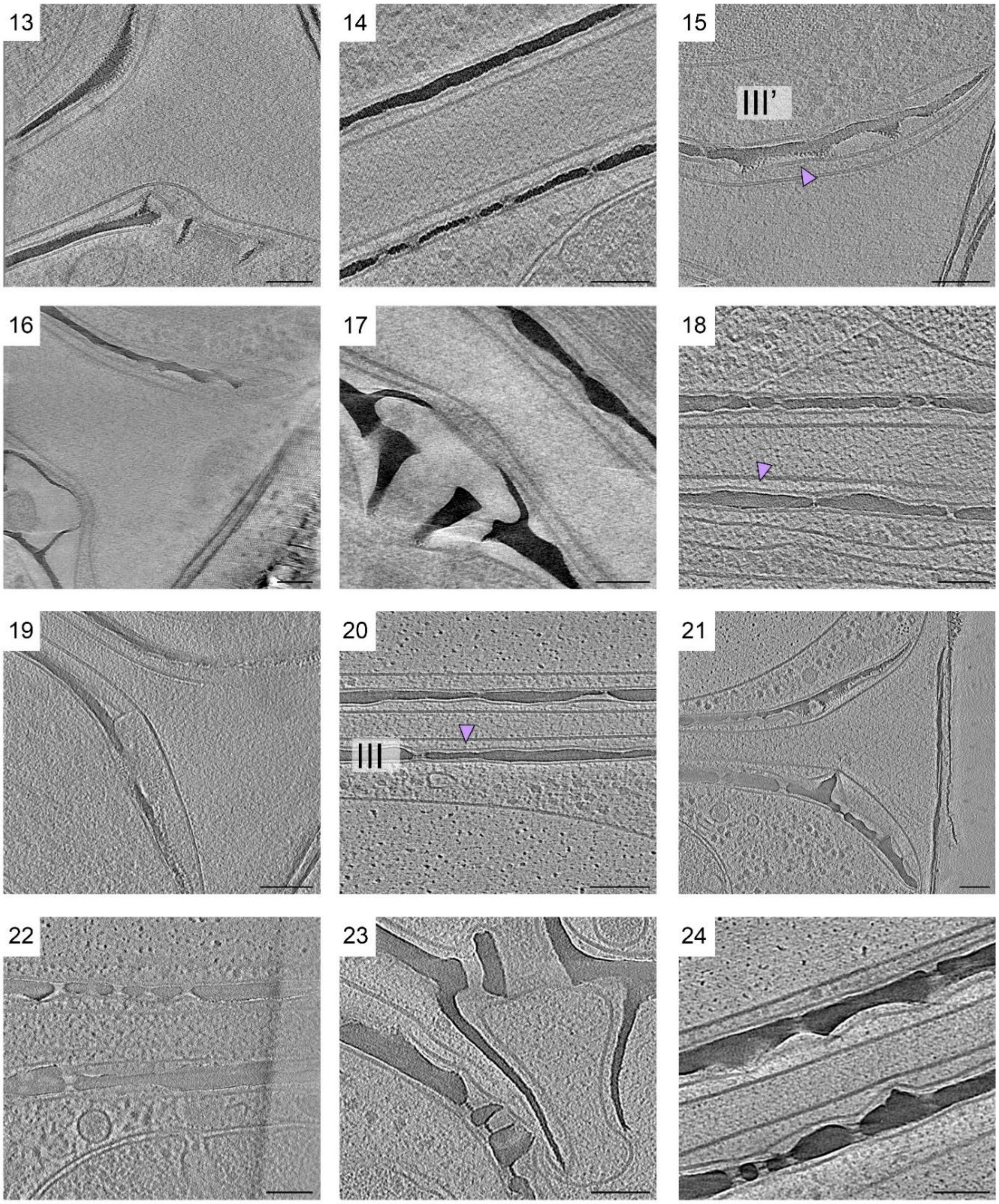

Figure S3 – Continued. Plate 2 out of 3.

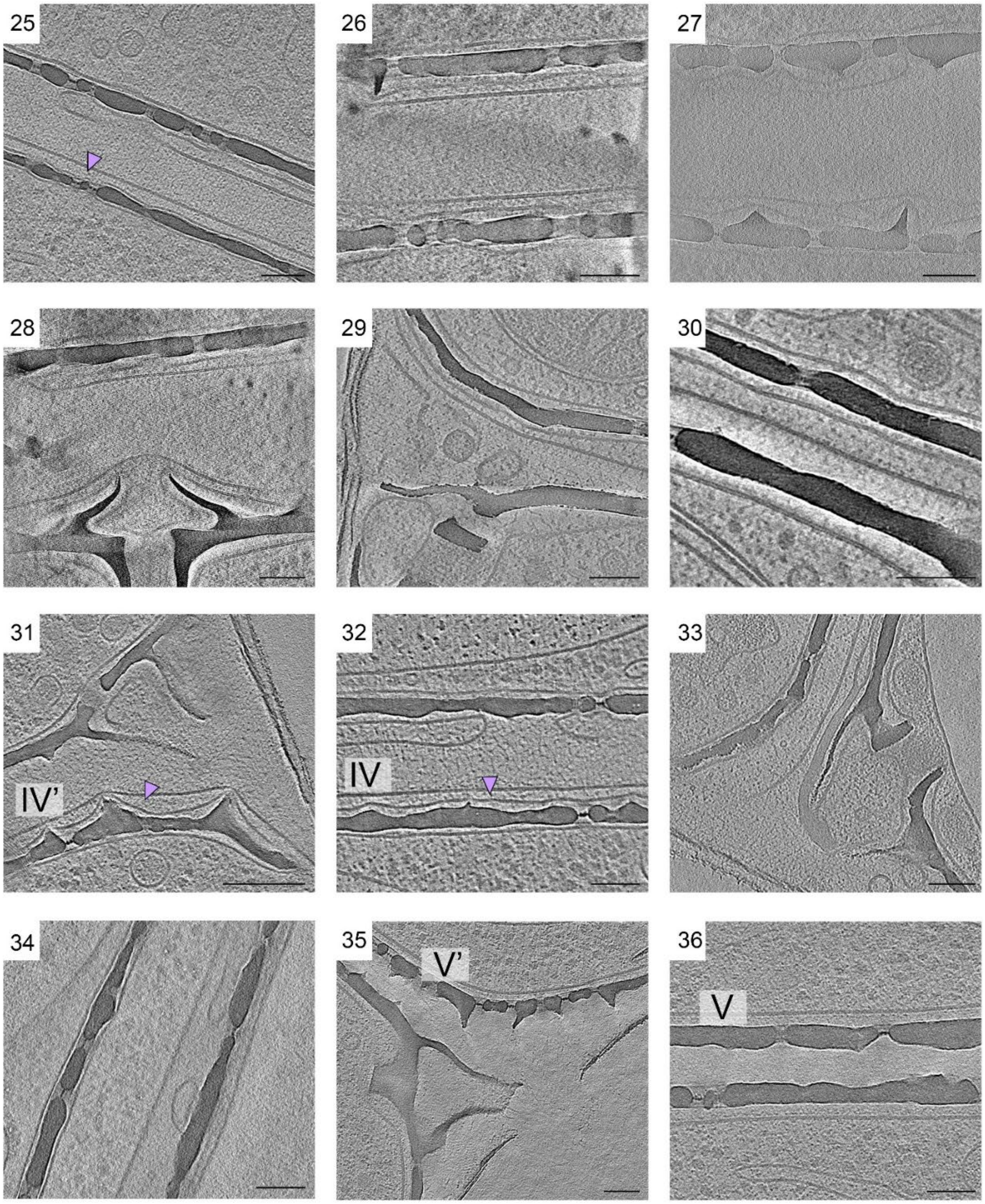
25
26
27
28
29
30
31
IV'
32
IV
33
34
35
V'
36
V

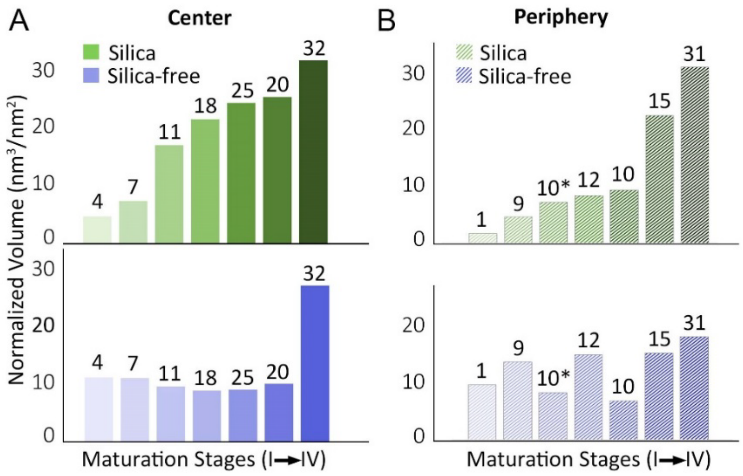

Figure S3 – Continued. Plate 3 out of 3.

The bar graphs at the end are the same as in Fig. 3, with a complete legend that explains which dataset was used to generate each bar in the datasets containing the center of the SDV (A), and periphery of the SDV (B). The numbers above the bars indicate the dataset number in this figure. Datasets 7, 11, 20 and 32 are the datasets of stages I, II, III and IV, respectively. Datasets 1, 10, 15 and 31 are the datasets of stages I', II', III' and IV', respectively. Source data are provided as a Source Data file.

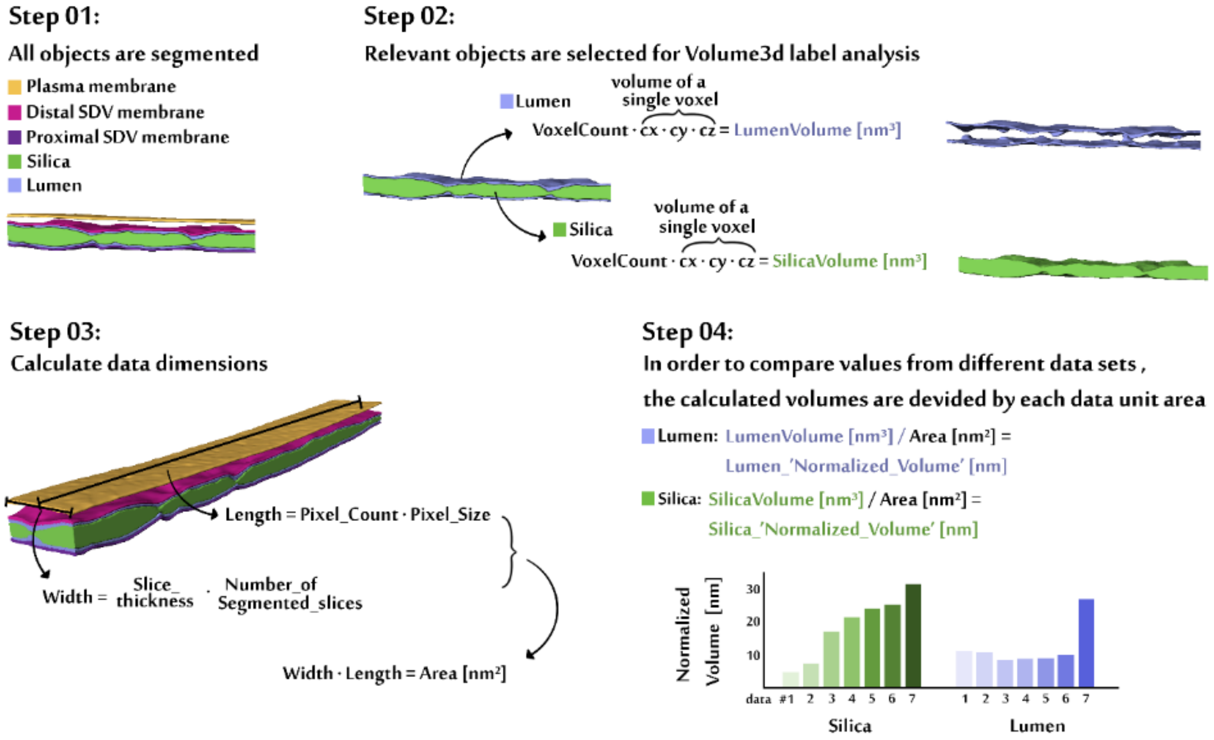

Figure S4 – Volume normalization workflow.

The calculation pipeline with visual examples illustrates the methodology used to compare the calculated volumes of the silica and the non-silicified SDV lumen. Since we had to normalize for the different sizes of the datasets, the reported value is the volume (in nm^3) per surface area unit of the SDV (in nm^2). To that end, we took advantage of the similarity between the shape of the valve and a thin slab (a box where height << length\width). The base area of this slab, namely the part of the valve present within each dataset, was used to normalize the volume to an 'area unit of the SDV slab'.

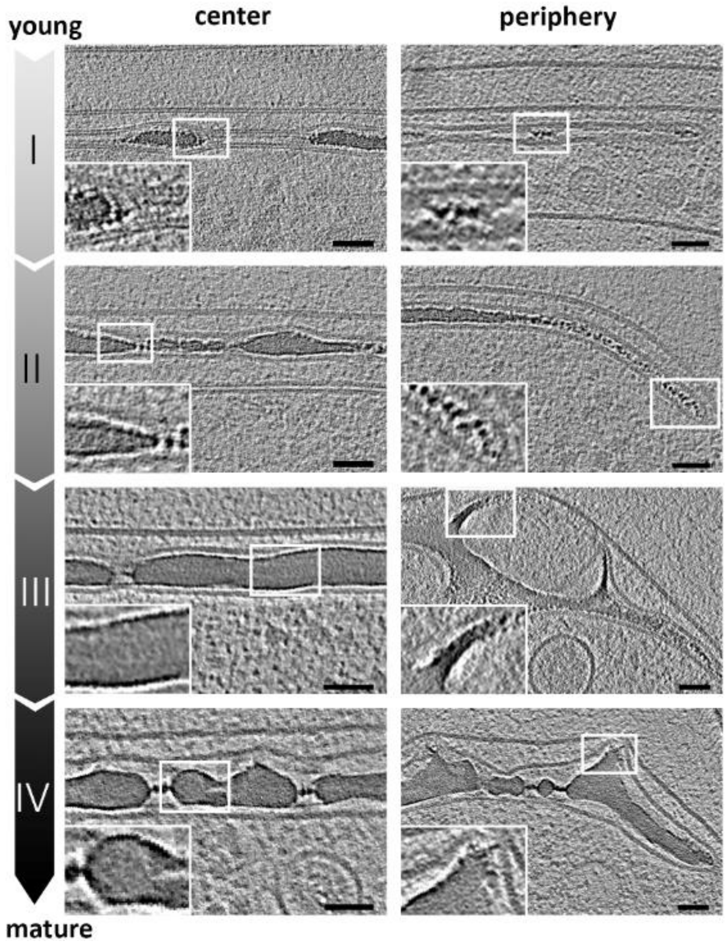

Figure S5 – Silica texture is granular at the initial stage.

High magnification of cryoET slices from four intracellular stages of silica formation (four at the SDV center and four at its periphery). During stages I and II, a granular texture dominates the silica, and also, in stages III and IV, such granular texture is visible at the developing elements at the periphery of the valve. Insets show high magnification of the areas marked by the rectangles. Scale bars are 50 nm.

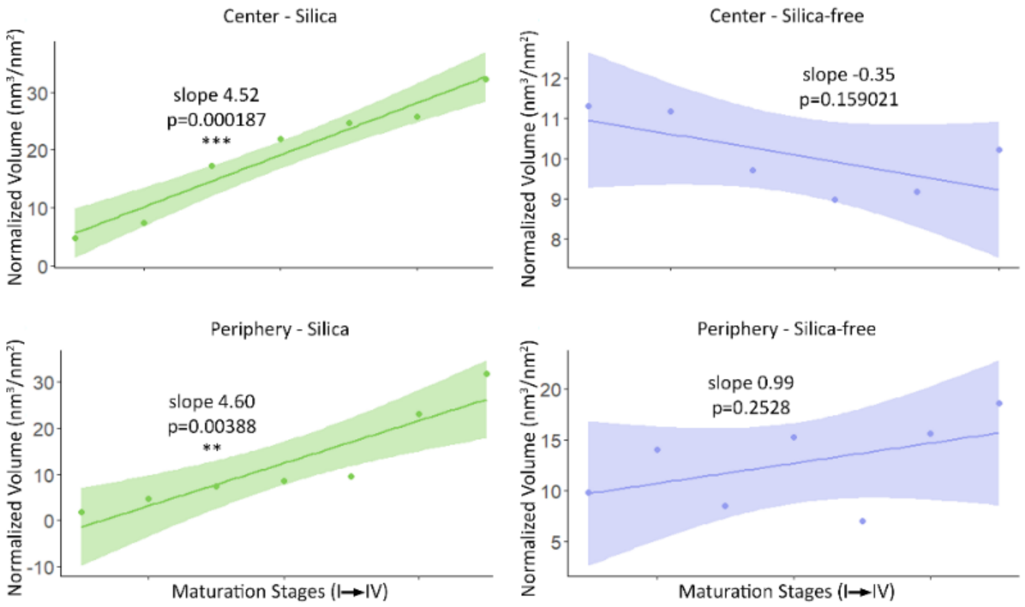

Figure S6 – Linear regression analysis of Fig. 3A

Linear regression lines (solid lines) are shown with the 95 % confidence interval (shading), plotted using the ‘geom_smooth’ function with method “lm” of the R package ‘ggplot2’. Source data are provided as a Source Data file.

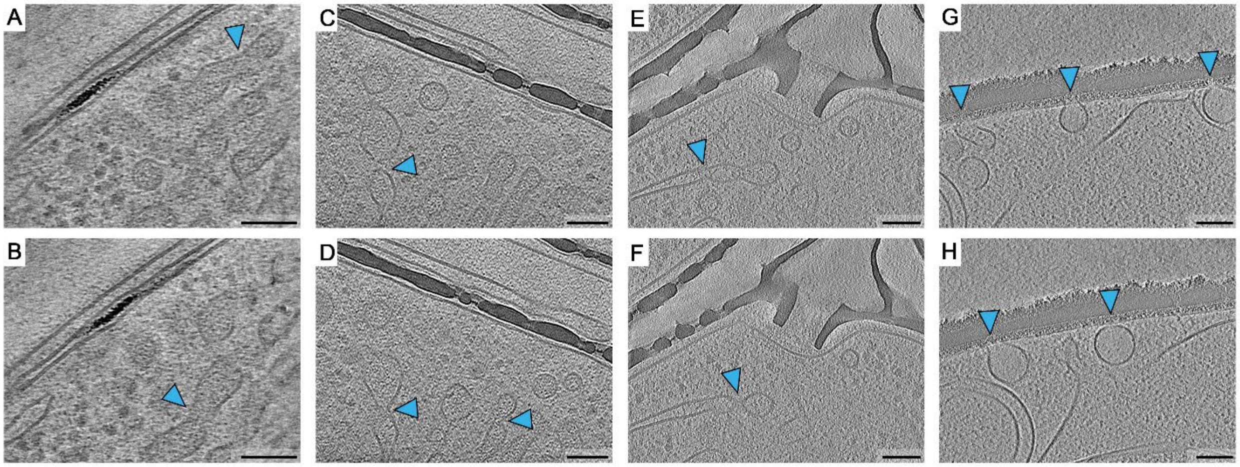

Figure S7 – Vesicle fusion events are detected in the datasets but not in association with the SDV membrane.

Eight representative slices of four cryoET datasets (two adjacent slices are shown from each dataset) show vesicle fusion (arrowheads). In panels A-F, the vesicle is near an SDV and Golgi. In panel G-H, vesicles are fused with the plasma membrane. Scale bars are 100 nm.

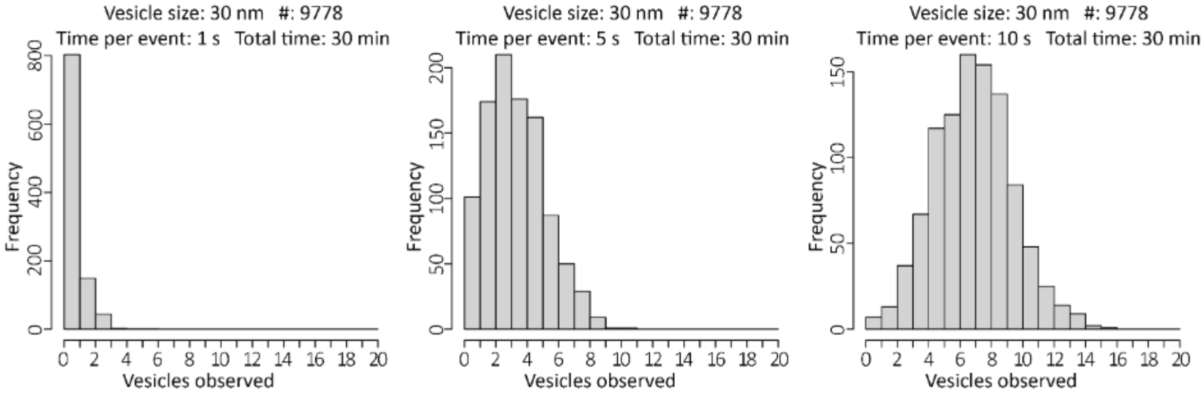

Figure S8 – modeling vesicle fusion probability.

Histograms show how many vesicle fusion events were observed in 1000 simulations for three different conditions. The simulation accounts for known quantitative values of some factors (maximal diameter and width of the SDV, time of valve formation, volume fraction of the SDV that is present in the thickness of the lamella) and, due to lack of information, uses putative values for other factors (size of the fusing vesicle and time of a fusion event), and reports on how many fusion events are expected to be seen in 50 datasets (similar to our data). The most important factor that skews the simulation is the unknown duration when

a fusion event is observable. If such putative fusion takes a second (left histogram), it will most likely not capture a single event in our datasets. However, if such an event takes 5 or 10 seconds (middle and right histograms), we should have seen at least 1 event with a probability of $p=0.890$ or $p=0.995$, respectively. Therefore, without prior knowledge of the simulated mechanism, it is difficult to assess its likelihood of participating in SDV formation.

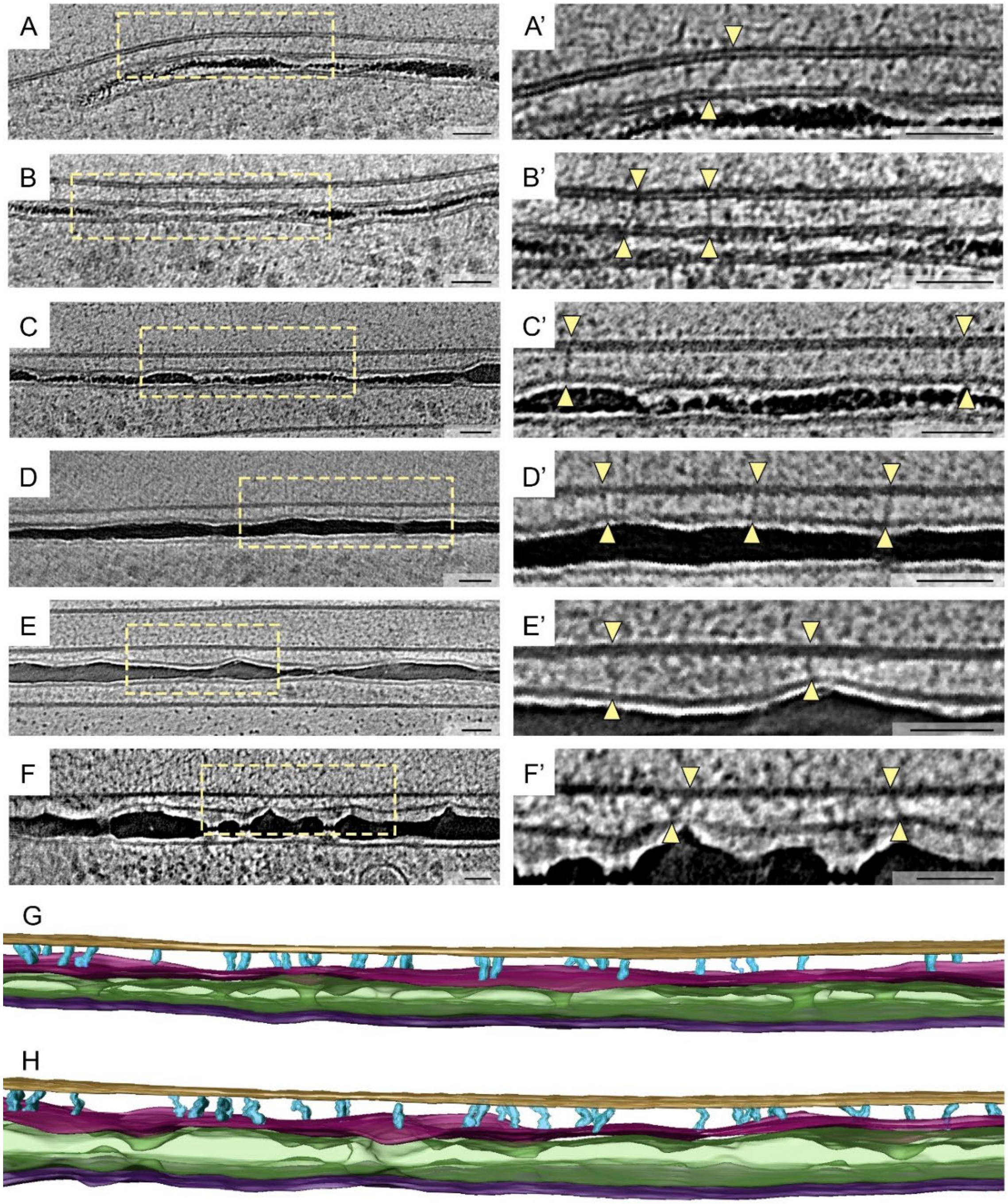

Figure S9 – Imaging contact sites between the SDV and the plasma membrane.

A-F) single slices out of denoised datasets of forming SDVs (C and F are the dataset of stages II and IV in Fig. 2, respectively). A'-F') high magnification images of A-F show contact sites between the distal SDV and plasma membranes. Scale bars are 50 nm. G-H) representative contact site segmentations of stages II and III.

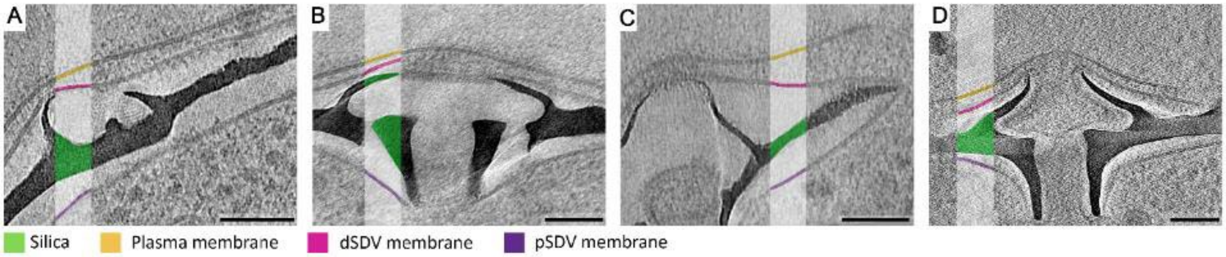

Figure S10 – Formation of fultoportulae within the SDV.

Four representative slices (A-D) from cryoET datasets that contain a forming fultoportula. The SDV membrane engulfs the entire structure but does not tightly line the intricate details of the fultoportula. Scale bars are 100 nm.

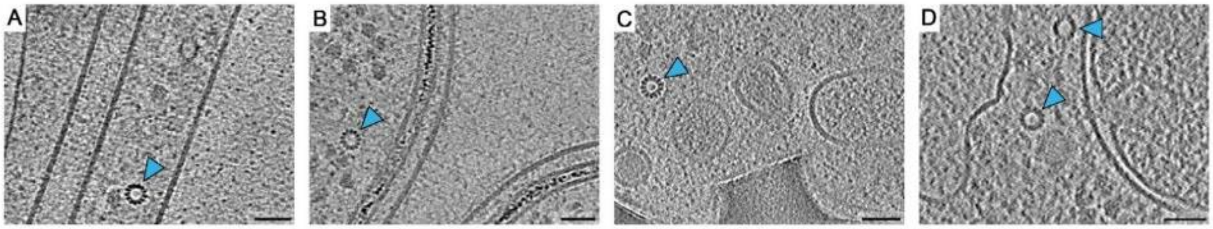

Figure S11 – Microtubules are easily detected in the datasets.

Four representative slices (A-D) from cryoET datasets that contain microtubules (arrowheads). In panels A, B and C, the microtubules are in the vicinity of an SDV (few hundreds of nanometers) but have no conserved association with it. Scale bars are 50 nm.

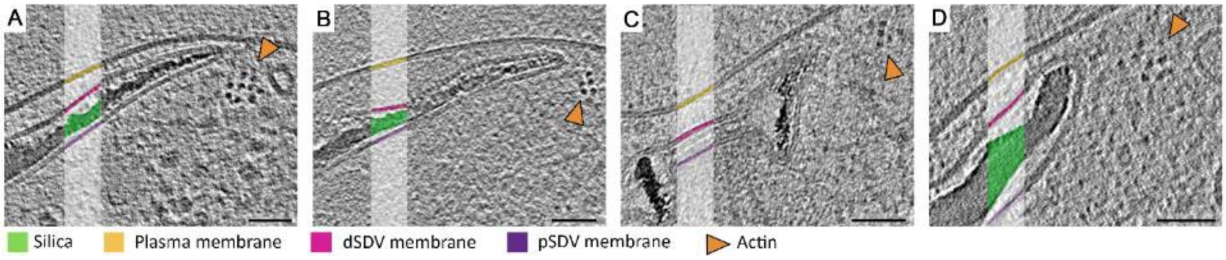

Figure S12 – The presence of actin bundles at the SDV periphery.

Four representative slices (A-D) from cryoET datasets that contain an expanding SDV. In all of them, a bundle of filaments is observed close to the expanding edge of the SDV (indicated with orange arrowheads). Scale bars are 50 nm.

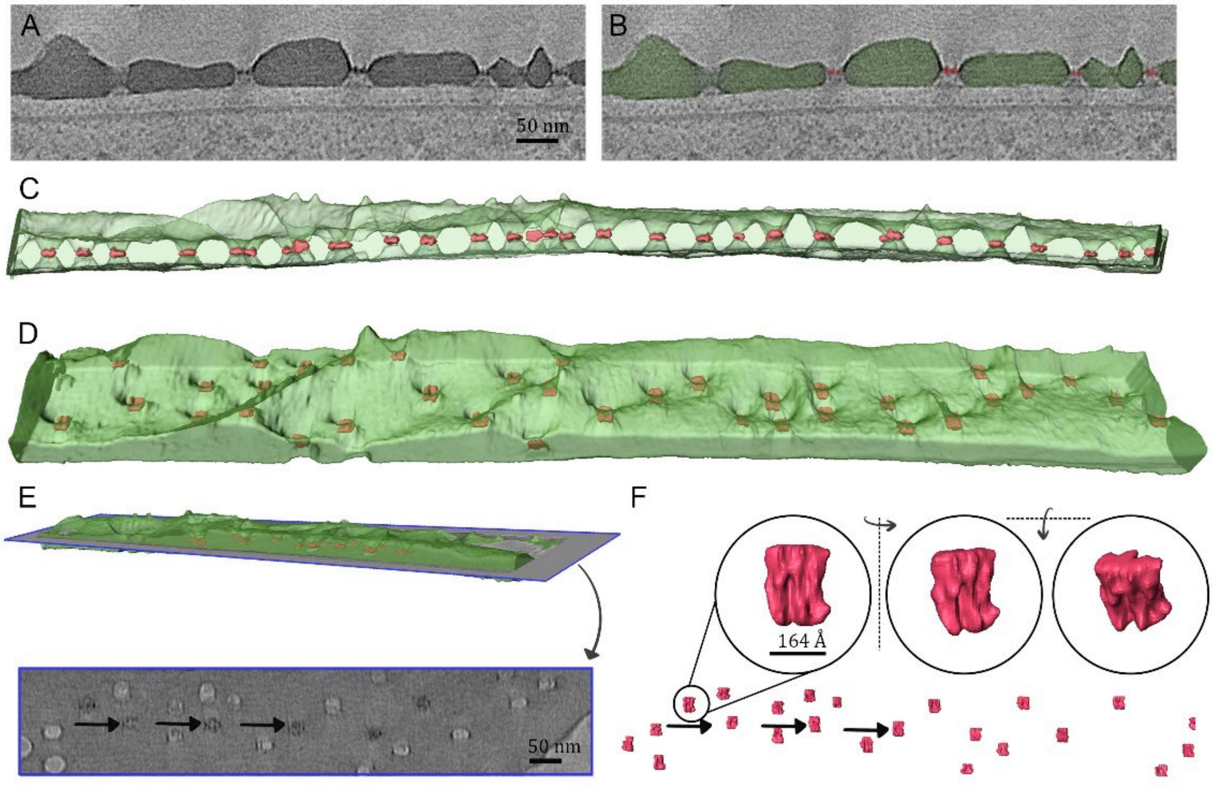

Figure S13 – The structure in the nanopores as observed with cryoET. A) Single slices through the reconstructed volume of a mature valve after exocytosis (stage V). B) same slice as in A with segmentation illustrating the different materials: silica in green, nanopore structure in red. C-D) The surface representation of the segmented data in A shows silica and nanopores. E) Top view of the silica nanopores. F) Surface representation of nanopore structure.

Table S1 - Regression statistics of SDV volumes in Fig. 3A and S6. The linear relationship between growth stage (predictor variable) and volume (outcome variable) were analysed by performing a simple linear regression using the lm() function in R.

Volume	Adjusted R2	F	df	Intercept	Slope	P-value
Center - silica	0.94	96.27	1, 5	1.10	4.52	0.00019
Center - silica free	0.28	2.99	1, 4	11.31	-0.35	0.15902
Periphery - silica	0.80	25.66	1, 5	-6.02	4.60	0.00388
Periphery - silica free	0.10	1.67	1, 5	8.73	0.99	0.2528

Table S2 - The Cliff's Delta values describe the overlap between membrane distance datasets in Fig. 4D. Overlap between groups is calculated as a value between 0 and 1.

	CD.m	
Stage comparisons	dSDV	pSDV
I - II	0.176	0.819
I - III	0.032	0.990
I - IV	0.138	1.000
II - III	0.138	0.670
II - IV	0.040	1.000
III - IV	0.104	0.999

Table S3 - Two-way ANOVA, testing the combined effect of membrane type and growth stage on the planarity in Fig. 4E. There is a highly significant effect of membrane type on planarity ($F_{2,6}$ = 66.436, $p <$ 0.0001), and a moderately significant effect of growth stage ($F_{3,6}$ = 7.258, p = 0.0202). A post-hoc TukeyHSD test to compare the differences in the planarity of the three membranes while accounting for

multiple comparisons, shows that the distal SDV membrane is less planar than both the proximal SDV membrane (p=0.0007) and plasma membrane (p<0.0001).

Membrane comparisons	P value
dSDV-PM	0.0000703 ***
pSDV-PM	0.0217741 *
pSDV-dSDV	0.0006848 ***

Simulation code with annotations

```
real.samples <- 60                # Number of SDVs observed
real.catches <- 0                 # Number of vesicles observed fusing to the SDV

# 1. Surface area of the mature valve SDV membrane ≈ surface area of a cylinder with diameter of 4 µm
and height of 200 nm
SDVh <- 0.2                       # height of the SDV
SDVr <- 2                         # radius of the SDV
SAsdv <- (2*pi*SDVh*SDVr)+(2*pi*SDVr^2) # Calculate the membrane surface area of the SDV

# 2. Membrane surface area of putative transport vesicles, with radius 0.0075,  0.015 or  0.04 µm
STVr <- 0.015                     # radius of the vesicle
SAstv <- 4*pi*STVr^2              # Calculate the membrane surface area of the vesicle

# 3. Calculation of the number of membrane vesicles required to form the entire SDV membrane
events <- SAsdv/SAstv

##Chance of observing putative vesicles in cryo-electron tomogram
# 4. Temporal aspect
event_time <- 10          # Duration of a fusion event for a vesicle of 30 nm: 0.1,  1  or 10 seconds
total_time <- 1800        # Total duration of SDV expansion: 1800  or 3600 seconds
temporal <- total_time/event_time      # Temporal chance of observing putative vesicles

# 5. Spatial aspect
Asdv <- pi*SDVr^2                 # Area of the SDV = where vesicles could fuse
Aslice <- 1.5*0.2                 # Area of a tomographic slice
Avesicle <- pi*STVr^2             # Area of a putative vesilce
spatial <- 1/(Avesicle/Asdv)*(Aslice/Asdv)     # The chance of catching a vesicle in a tomogram

success1 <- numeric(length=1000)
```

```
# 6. A loop
for (i in 1:1000) {                              # Number of iterations
  if (i %% 100 == 0) message(i)                  # Print progress at every 100th iteration
  one.round <- numeric(length=real.samples) # Create an empty vector to save the outcome of each iteration of the next loop
  for (j in 1:length(one.round)) {              # A nested loop with one iteration for each observed SDV
    one.cell <- matrix(0, nrow=events, ncol=2)  # Create a dataframe with a row for each putative vesicle as determined in step 3.
    one.cell[,1] <- sample(1:temporal, nrow(one.cell), replace=TRUE) # Random generation of catching each putative vesicle based on the temporal aspect
    one.cell[,2] <- sample(1:spatial, nrow(one.cell), replace=TRUE) # Random generation of catching each putative vesicle based on the spatial aspect
    for (k in 1:nrow(one.cell)) {               # A nested loop with one iteration for each putative vesicle
      if (one.cell[k, 1] == 1 & one.cell[k, 2] == 1) { # If the putative vesicle was caught based on both the temporal and spatial chance, record it as succes
        one.round[j] <- 1
      }
    }
  }
  success1[i] <- sum(one.round)     #Write the number of successes per loop
}

# print the results
hist(success1, main = paste("Vesicle size:",STVr*2*1000,"nm   #:",round(events,0),"\nTime per event:",event_time,"s  Total time:",total_time/60,"min"),xlim = c(0,20),breaks = 20,xlab="Vesicles observed",xaxt='n')
axis(side=1, at=seq(0,20, 1), labels=seq(0,20,1))
```
