# Supplementary material for: Intracellular morphogenesis of diatom silica is guided by local variations in membrane curvature
Source: Nat Commun. 2024 Sep 10;15:7888. doi: 10.1038/s41467-024-52211-x (PMC11385223; doi:10.1038/s41467-024-52211-x)
Supplement: Supplementary file 3 — Description of Additional Supplementary Files [file 41467_2024_52211_MOESM3_ESM.pdf]

Description of Additional Supplementary Files

Supplementary Movie Legends

Supplementary Movie 1. Animation of the analyses made on the cryo-ET dataset of SDV in Stage I, shown in Figures 2,4,5.

Supplementary Movie 2. Animation of the analyses made on the cryo-ET dataset of SDV in Stage II, shown in Figures 2,4,5.

Supplementary Movie 3. Animation of the analyses made on the cryo-ET dataset of SDV in Stage III, shown in Figures 2,4,5.

Supplementary Movie 4. Animation of the analyses made on the cryo-ET dataset of SDV in Stage IV, shown in Figures 2,4,5.
